# King Edward's Hospital Fund for London

**Published:** 1907-12-21

**Authors:** 


### HOSPITAL ADMINISTRATION.

### CONSTRUCTION AND ECONOMICS.

### KING EDWARD'S HOSPITAL FUND FOR LONDON.

DISTRIBUTION MEETING.

A meeting of the President and General Council of King Edward's Hospital Fund for London, for the purpose of awarding grants to the hospitals and convalescent homes for the present year, was held on December 16, at Marlborough House, the Prince of Wales being in the chair. An official report of the proceedings which has been supplied to us is to the following effect:—

Those present were: The Duke of Norfolk, the Bishop of London, the Rev. T. Bowman Stephenson, D.D., the Chief Rabbi (the Rev. Dr. Adler), Lord Iveagh, Lord Rothschild, Lord Farquhar, the President of the Hospital Sunday and Hospital Saturday Funds (the Lord Mayor), the Chairman of the County Council (Mr. H. Percy Harris), the Governor of the Bank of England (Mr. W. Middleton Campbell), the President of the Royal College of Physicians (Sir R. Douglas Powell), the President of the Royal College of Surgeons (Mr. Henry Morris), Sir Savile Crossley, Sir Joseph Dimsdale, Mr. C. Stuart Wortley, K.C., M.P., Sir Trevor Lawrence, Sir W. S. Church, Sir Julius Wernher, Sir Henry Burdett, Sir W. J. Collins, M.P., Sir John Craggs, Mr. Frederick M. Fry, Mr. William Latham, K.C., Mr. J. Danvers Power, Mr. Albert G. Sandemann, and Mr. Hugh C. Smith. There were also present: Lord Revelstoke, the President of the Local Government Board (Mr. John Burns, M.P.), the Master of Elibank, M.P., Sir Ernest Cassel, Lieutenant-Colonel Sir Arthur Bigge, Sir Horace Marshall, Dr. Freshfield, Mr. John G. Griffiths, and Mr. F. Vaughan Hawkins.

Lord Rothschild reported that the amount received by the Fund to December 12, after payment of expenses, was £147,263 2s. 7d.

Sir Henry Burdett stated that, although this year had been a very bad year for the League of Mercy, owing to the general depression and the difficulty of raising small sums even from so many people, yet he was pleased to say the actual results were better than in any previous year. They would, therefore, be able to give at least the same grant to the King's Fund this year as last year—namely, £18,000. (Hear, hear.)

The Chairman of the Executive Committee (Mr. Hugh Colin Smith) read the report of the Executive Committee.

The Chairman of the Hospitals Distribution Committee (Sir William Church) read the report of that committee.

Awards for 1907.

Sir Savile Crossley presented the list of awards as follows:—

The Distribution Committee desire to draw attention to the fact that the absence of a grant does not necessarily imply dissatisfaction. [In the following list the committee's "remarks," where any are made, follow the amount of the grant in each case.]

Alexandra Hospital for Children.—£500.
Belgrave Hospital.—£1,500; of which £1,000 to reduce maintenance debt.
Blackheath and Charlton Cottage Hospital.—£100.
Bolingbroke Hospital.—£3,000; of which £2,500 to reduce building debt.
British Lying-in Hospital.—£350; of which £150 to reduce debt.
Cancer Hospital.—No grant; the committee are glad to see that no assistance from the Fund is required.
Central London Ophthalmic Hospital.—£100; to bedsteads and bedding.
Central London Throat and Ear Hospital.—No grant; see paragraph 4 of report.
Charing Cross Hospital.—£3,000.
Chelsea Hospital for Women.—£500.
Cheyne Hospital for Sick and Incurable Children.—£50; in consideration of the fact that some curable cases are admitted.
City of London Hospital for Diseases of the Chest, Victoria Park, E.—£2,500.
City of London Lying-in Hospital.—£250.
Clapham Maternity Hospital.—£100.
Dreadnought Hospital.—£2,500.
East-end Mothers' Lying-in Home.—£500; of which £350 to alterations.
East London Hospital for Children.—£2,000; of which £1,000 to improvements.
Eltham and Mottingham Cottage Hospital.—£50; to improvements.
Evelina Hospital.—£1,250; of which £1,000 to reduction of out-patient building debt.
French Hospital.—£200.
Friedenheim Hospital.—£50.
General Lying-in Hospital.—£250.
German Hospital.—£200.
Gordon Hospital for Fistula, &c.—£25.
Great Northern Central Hospital.—£2,000; of which £800 to complete purchase of land.
Grosvenor Hospital for Women and Children.—£250.
Guy's Hospital.—£15,000; of which £10,000 is an exceptional grant to enable the new out-patient department to be taken in hand during the present winter.
Hampstead General Hospital.—No grant; an exceptional grant of £5,000 was given in 1906.
Home and Infirmary for Sick Children.—£250; of which £100 to reduce debt on mortuary.
Hospital for Consumption.—£2,500; the committee view with special satisfaction the work done by this institution in its country sanatorium.
Hospital for Diseases of the Throat.—No grant; see paragraph 4 of the report.
Hospital for Epilepsy and Paralysis.—£1,000; of which £500 to improvements, including an operating room.
Hospital for Invalid Gentlewomen.—£1,000; an exceptional grant towards building fund.
Hospital for Sick Children.—£1,750.
Hospital for Women.—£3,000; of which £2,000 to rebuilding.
Hospital of St. John and St. Elizabeth.—£500.
Italian Hospital.—£500.
Kensington Dispensary and Children's Hospital.—£25.
Kensington General Hospital (late Queen's Jubilee Hospital).—No grant.
King's College Hospital.—£7,000; of which £6,000 to Removal Fund.
London Fever Hospital.—£150.
London Homœopathic Hospital.—£550.
London Hospital.—£10,000.
London Lock Hospital.—£1,100; of which £600 to nursing accommodation. The committee will be prepared to recommend a substantial great towards the expense of rebuilding the hospital at Dean Street, when a definite scheme has been submitted and approved.
London Temperance Hospital.—£1,600; of which £1,000 to out-patient debt.
London Throat Hospital.—No grant; see paragraph 4 of report.
Maternity Charity and District Nurses' Home, Plaistow, E.—£50.
Medical Mission Hospital (formerly entered as Cottage Hospital, Canning Town), Balaam Street, Plaistow.—£300.
Memorial Cottage Hospital (Mildmay Park).—£25.
Metropolitan Hospital.—£2,500; the committee are pleased to see the successful efforts being made to reduce expenditure.
Middlesex Hospital.—£3,000.
Mildmay Mission Hospital.—£275.
Miller Hospital (Greenwich).—£2,000; of which £1,250 to extension, provided the scheme is for a total of not more than 50 beds.

Mount Vernon Hospital for Consumption (formerly entered as North London Hospital for Consumption).—£1,000; an exceptional grant of £4,500 was made in 1906.

National Anti-Vivisection Hospital.—No grant.

National Dental Hospital.—£150; to reduce debt.

National Hospital for the Paralysed and Epileptic.—£2,000.

New Hospital for Women.—£1,000; of which £250 to electric light.

North-Eastern Hospital for Children.—£2,000; of which £1,000 to building debt.

North-West London Hospital.—£250; in consideration of the work done by the hospital pending the decision as to its future.

Norwood Cottage Hospital.—£150; of which £100 to improvements.

Paddington Green Children's Hospital.—£750; of which £250 to reduce debt. The committee view with satisfaction the efforts made to reduce expenditure.

Passmore Edwards Acton Cottage Hospital.—£50.

Passmore Edwards East Ham Hospital.—£50.

Passmore Edwards Hospital for Willesden.—£25.

Passmore Edwards Cottage Hospital, Wood Green.—No grant.

Poplar Hospital.—No grant; a well-managed economical institution.

Prince of Wales's General Hospital.—£2,500, of which £500 to reduce debt.

Queen Charlotte's Lying-in Hospital.—£500.

Royal Dental Hospital of London (formerly entered as Dental Hospital).—£500.

Royal Ear Hospital.—No grant; see paragraph 4 of report.

Royal Eye Hospital.—£600; the committee are glad to see the reduction in the cost per bed.

Royal Free Hospital.—£3,000, of which £1,000 to operating theatres.

Royal Hospital for Diseases of the Chest, City Road.—£1,000, an exceptional grant to open a fund for a country sanatorium. The committee consider that an open-air sanatorium in the country is essential for the consumptive patients of this hospital.

Royal London Ophthalmic Hospital.—£2,500.

Royal National Orthopædic Hospital.—£1,500, in accordance with amalgamation agreement.

Royal Waterloo Hospital for Children and Women.—£1,750, the balance required to close the building fund to be paid first out of this grant.

Royal Westminster Ophthalmic Hospital.—No grant; the committee are glad to see that the hospital does not require assistance from the Fund this year.

St. George's Hospital.—£2,500.

St. John's Hospital for Diseases of the Skin.—£100.

St. John's Hospital, Morden Hill, Lewisham, S.E.—£150.

St. Luke's House.—No grant.

St. Mark's Hospital.—£100.

St. Mary's Hospital, Paddington.—£3,500.

St. Mary's Hospital for Women and Children, Plaistow.—£1,500, of which £1,000 to building.

St. Monica's Hospital.—£150, of which £50 to verandah.

St. Peter's Hospital for Stone.—£50.

St. Saviour's Hospital, Osnaburgh Street.—£200.

Samaritan Free Hospital.—£750, of which £250 to improvements.

Santa Claus Home.—£25, in consideration of the fact that some curable cases are admitted.

South Wimbledon, Merton, and District Cottage Hospital.—No grant; the committee would be prepared to recommend a grant towards rebuilding when an approved scheme is set on foot.

University College Hospital.—£3,000; the committee are glad to see the reduction in the cost per bed.

Victoria Hospital for Children.—£1,250, of which £250 to reduce bank debt.

West End Hospital for Diseases of the Nervous System.—£250.

Western Ophthalmic Hospital.—£150, to reduce debt.

West Ham and East London Hospital.—£1,750, of which £1,250 to building.

West London Hospital.—£3,000, of which £1,500 to reduce bank debt.

Westminster Hospital.—£3,000.

Woolwich and Plumstead Cottage Hospital.—No grant.

SPECIAL GRANT

Set aside for amalgamation of throat and ear hospitals ... ... ... £2,000

SUMMARY.

Grants ... ... ... ... ... ... £118,000
Special grant set aside for amalgamation of throat and ear hospitals 2,000

Total grants to hospitals for the year 1907 ... ... ... £120,000

The Chairman of the Convalescent Homes Committee (Mr. William Latham, K.C.) read the report of that committee as follows:—Owing to the continued liberality of the Trustees of the London Parochial Charities the committee have again £1,000 to distribute. The committee have continued their practice of giving the preference to institutions directly connected with London hospitals. The following awards are recommended:—

Alexandra Hospital, Convalescent Home at Painswick.—£25.

Charing Cross Hospital, Convalescent Home at Limpsfield, Surrey.—£50.

Chelsea Hospital for Women, Convalescent Home at St. Leonards.—£25.

East London Hospital for Children, Convalescent Home at Bognor.—£100.

Hospital for Sick Children, Convalescent Branch, Cromwell House, Highgate, N.—£25.

London Hospital, Convalescent Home at Tankerton, near Whitstable.—£50.

Mary Wardell Convalescent Home, Stanmore, Middlesex.—£25.

Metropolitan Convalescent Institution, Convalescent Home at Walton.—£100.

Metropolitan Convalescent Institution, Convalescent Home at Broadstairs.—£100.

Middlesex Hospital, Convalescent Home at Clacton-on-Sea.—£200.

Mildmay Mission Hospital, Convalescent Home at Hendon.—£50.

Mrs. Gladstone's Free Convalescent Home for the Poor, Ravensbury House, Mitcham, Surrey.—£50.

Mrs. Kitto's Free Convalescent Home, South Park, Reigate.—£25.

National Hospital for the Paralysed and Epileptic, Convalescent Home at East Finchley.—£25.

Paddington Green Children's Hospital, Convalescent Home at Slough.—£25.

Queen Charlotte's Lying-in Hospital, Convalescent Home at 20 Victoria Road, Kilburn, N.W.—£25.

Victoria Hospital for Children, Convalescent Home at Broadstairs.—£100.

Total Grants to Convalescent Homes.—£1,000.

### Speech by the President.

The Prince of Wales, in moving the adoption of the reports and awards, said:—In moving the adoption of the reports, I ought first to mention that the receipts for 1907 have included some very large sums, such as the munificent gift of £100,000 from Mr. Carnegie, which will probably be placed to capital. We have also had exceptional sums from legacies; but even in this specially fortunate year it seems advisable not to distribute more than the average amount of the legacies received in past years, which has hitherto been our custom. The sum recommended for distribution to hospitals this year has, I am pleased to say, reached £120,000. (Hear, hear.) There are one or two items in the grants which require a word of explanation. A very exceptional sum of £15,000 has been allocated to Guy's Hospital, and I cannot help thinking that the policy of occasionally making such grants is a sound one, and one which I trust will not be misunderstood. The most important work of rebuilding the out-patient department was being delayed, to the detriment of patients in a very poor part of London, for the want of £10,000. The King's Fund, in any case, would have ultimately contributed as much as £10,000 to this object, but the contributions would have probably been spread over several years. Instead of this, we propose to pay the whole sum now, and the work will be carried out at once. I hope that this will be of some benefit to those who are employed upon the building during the winter months, as well as to those for whom the building is intended. (Hear, hear.) We shall naturally remember for some little time to come that Guy's had this exceptional grant in 1907. As you will remember, we gave £5,000 last year to the Hampstead General Hospital in order to enable it to take advantage of a conditional offer of £20,000, and you will notice that no grant to that hospital has been recommended this year. We propose to give a considerable sum to the removal fund of King's College Hospital, and the grant of £6,000 will make our total contributions up to £22,000 to this object. A matter which I am sure you will agree ought to engage our most serious attention in the future is that relating to the treatment of consumption, and I am glad to see that the Distribution Committee in the awards to consumption hospitals are giving special attention to the necessity for sanatoria. Whether this subject should be treated as distinct from the ordinary hospital work of London I need not at present discuss; but I feel sure that the want of consumption sanatoria in the country, for London patients, is very great, and I think that the King's Fund should do all in its power to encourage the establishment of these useful institutions. (Hear, hear.) Another question for consideration is that which relates to the publishing in our reports of the names of hospitals which, having applied, yet do not receive any grant. I hope the Distribution Committee will carefully weigh the matter in all its bearings and advise us what ought to be done. The system which was adopted last year of not paying grants for special purposes until the occasion for expending the money actually arises has appeared to work very satisfactorily. In order to avoid money being left idle indefinitely, special grants are now always accompanied by the condition that the Fund shall have an opportunity of reconsidering the matter in two years if the money has not been spent on the object for which it was given. For some years we have had occasion to notice that certain hospitals do not issue their annual reports and accounts until rather late in the following year, and I think our Committee have done wisely to propose that these should be issued not later than April 15 in future. It does not seem unreasonable to expect that three-and-a-half months is sufficient time for any hospital to prepare its annual statement to the subscribers. The whole of the useful information which is provided in our statistical report may be delayed by a single institution being behindhand in this respect. Turning to other work which has engaged our attention during the past year, I may pass over the important event in the history of the Fund of our having obtained an Act of Parliament, which places the Fund on a legal basis, as I have already referred fully to that on a previous occasion. The amalgamation of the orthopædic hospitals is fortunately complete, and there is now only one orthopædic hospital, which, I trust, will worthily take its place among the special hospitals of London. The efforts to promote an amalgamation between the five throat and ear hospitals are continuing, and I trust they may ultimately be successful. The Fund has made a liberal offer to the Hampstead General Hospital, provided it takes over the work of the North-West London Hospital on certain conditions, and we hope to be able to report favourably on this next year also. The only other subject to which I wish to call your attention is that of the Workmen's Compensation Act and insurance companies. I am aware of the difficulties which insurance companies have to deal with in the matter of subscriptions to charities and institutions which affect the risks they take. The premiums charged must naturally be based on all surrounding circumstances, among which the existence of hospitals is, I suppose, an important factor. In conclusion, I have to thank the League of Mercy for the great sum which Sir Henry Burdett has just announced, and which is the result of their continued persevering efforts. We are also indebted to the Trustees of the London Parochial Charities for their annual grant of £1,000 for convalescent homes. I desire to thank Sir Eyre Shaw and Captain Wells for their valuable report on fire prevention, which we have circulated among the hospitals for their consideration, and, as doubtless will be found, for their assistance. To all the honorary officials of the Fund, and to those who have served on the Council and various committees, I offer my warmest thanks, as well as to the honorary officers of the Fund, whose work this year has been unusually heavy, in consequence of the business connected with our Act. And here I must refer with great regret to the retirement of one of our honorary secretaries, Mr. Danvers Power, whose services cannot be spoken too highly of. (Hear, hear.) He has compiled our statistical report—in itself an enormous asset to the Fund—and his qualities as a man of business, and of practical experience in the hospital world, are too well known to all of us to need comment. However, I am glad to think we shall retain him on our Council and Executive, and to know that his advice will always be available, and his interest undiminished. That Mr. Frederick Fry has consented to take up his work is a great mitigation of the loss; there is no one who could so well fill his place. (Hear, hear.) I now move the adoption of the reports. (Hear, hear.)

Mr. John Burns, in seconding the adoption of the reports, said he wished to associate himself heartily with his Royal Highness's remarks about Mr. Danvers Power, of whose work he had himself had experience in connection with the Queen's Unemployed Fund. As President of the Local Government Board it was his duty to nominate an auditor, and he suggested that the interests of the Fund would be best served if the present honorary auditors (Messrs. Deloitte, Plender, Griffiths, and Co.) continued in their position for next year, after which they would be better able to judge whether any alteration was necessary. He also wished to refer to the question of the relationship of general hospitals and Poor-law infirmaries, and outdoor relief generally. There was considerable overlapping, and he suggested that a committee of the Fund should look into the matter. In regard to consumption sanatoria there was a demand for the compulsory notification of consumption and tuberculosis. If this demand were hurriedly complied with, there would be an enormous, almost an overwhelming, claim made for new sanatoria. He had, as a result of the laborious industry of Dr. Bulstrode, obtained valuable information on this very important, almost tragic, question, and he proposed early next month to circulate it at home and abroad. In taking his seat as a member of the Council of the King's Fund he had the greatest pleasure in seconding the adoption of the reports.

The adoption of the reports was then put and carried unanimously.

On the motion of Mr. Hugh C. Smith, it was resolved that the cheques for the grants should be posted on December 19.

### Arrangements for 1908.

The President then said: It may be convenient if I now state to the Council what the probable arrangements for the next year will be. Under the Act I must appoint a new Council every year, and the appointment must be made in, and in respect of, the year in which the Council is to act. I cannot, therefore, nominate the Council for next year until January. It is my intention, however, to invite all who have hitherto so kindly given me their help to continue their services, and I have also been fortunate enough to secure the services of some new members, many of whom we have the pleasure of seeing here to-day. It will, I trust, be understood that, as the convenience of transacting business round a table which has its limits must be considered, it is not possible for me to add the names of many others to whom we are greatly indebted for services on the committees or as visitors. As, no doubt, the first work of the new Council will be the delegation of some of its powers to committees, and feeling that you will prefer that the matter should be discussed now, so that the meeting in the beginning of January need only be formal, I have directed the necessary resolutions to be prepared, and they lie on the table. You will, I think, agree with me that the work is satisfactorily divided by the existing committees. They are, as you know, the Executive, Finance, Distribution, and Convalescent Homes Committees, and our visitors. It will be my duty to nominate the members, and I should propose, in the first instance, to proceed on the principle of not nominating more than one gentleman to one committee. I think it will be necessary that the honorary secretaries should have the right to attend and speak at all committees without, however, voting on more than the one to which they belong. Time will show whether this arrangement will always meet our necessities; but I feel that we should endeavour, within workable limits, to give the fullest possible opportunity to the greatest number of taking part in the work of the Fund. (Hear, hear.) The visitors have hitherto been spoken of by us as a committee, but their duties, invaluable as they are to the Fund, are personal rather than corporate. I propose to appoint them as officials at a later date. The work of the committees, if you decide to delegate some of your powers to them, would, under the draft resolutions, be divided in this way: The Finance Committee will deal with all questions of investment. The Distribution Committee will advise the General Council as to grants to hospitals, deciding which hospitals shall be placed on the list for consideration, and making rules for the observance of the hospitals in connection with their applications. The Convalescent Homes Committee will advise as to the grants to convalescent homes and sanatoria beyond the metropolitan area. I have already referred to the importance of sanatoria for the treatment of consumption, and I do not think that the fact that many of these institutions are outside the area now visited by us should preclude us from considering their claims. It must not be forgotten that many of their inmates are patients from the London hospitals. I hope next year we may devote some portion of our funds in this direction. This question seems to me well worthy of your consideration. (Hear, hear.) With the exception of those duties assigned to the other committees, and reserved to the Council, the general work of the Fund will be entrusted to the executive committee. I hope to attend the meetings of the Council regularly, as hitherto. In case I should be prevented from doing so, it will, of course, be necessary that someone presides for me. I am not sure that it is my duty to nominate a substitute, but, at the same time, I should like to think that my place would be taken by Mr. Hugh Smith, who would bring his long experience as Chairman of the Executive to the service of the Council. (Hear, hear.) In regard to rules and regulations, I doubt whether in a body of this kind, where we are all animated by one object, it will be necessary to frame any standing orders. The Act of Parliament gives us our constitution in its broad lines, and it will, of course, be the desire of all of us to carry it out in the spirit as well as the letter. The draft resolutions appointing committees provide for the current work and define the powers of those committees.

The Distribution Committee, as I have said, will have power to make rules and regulations for the observance of institutions applying to us for grants. Under this Act we are subject to annual reconstruction. We know, therefore, that what is decided to be best now is not an immutable law for future generations, or even for the year after next. I now suggest that we should discuss these resolutions, and, if they are approved, record our approval for the guidance of the meeting in January, which will, doubtless, confirm our decision. Before doing so, however, I will read the names of the 1908 Council and committees which I intend to appoint:—

### Members of General Council.

#### Old Members.

The Lord Lieutenant of the County of London (the Duke of Fife), the Lord Lieutenant of Middlesex (the Duke of Bedford), the Duke of Norfolk, the Bishop of London, the Bishop of Southwark, Archbishop Bourne, the Rev. J. Guinness Rogers, D.D., the Rev. T. Bowman Stephenson, D.D., the Chief Rabbi (the Rev. Dr. Adler), Lord Bessborough, Lord Iveagh, Lord Rothschild, Lord Strathcona and Mount Royal, Lord Farquhar, the Postmaster-General, the President of the Hospital Sunday and Hospital Saturday Funds (the Lord Mayor), the Chairman of the County Council, the Governor of the Bank of England, the President of the Royal College of Physicians (Sir R. Douglas Powell), the President of the Royal College of Surgeons (Mr. Henry Morris), Sir Savile Crossley, Sir Joseph Dimsdale, Mr. Thomas Burt, M.P., Mr. C. Stuart-Wortley, K.C., M.P., Sir Trevor Lawrence, Sir John Aird, Sir W. S. Church, Sir Julius Wernher, Sir Edgar Speyer, Sir Henry Burdett, Sir W. J. Collins, M.P., Sir John Craggs, Mr. Frederick M. Fry, Mr. E. A. Hambro, Mr. William Latham, K.C., Mr. J. Pierpont Morgan, jun., Mr. J. Danvers Power, Mr. Albert G. Sandeman, Mr. Hugh C. Smith.

#### New Members.

The Chancellor of London University (Lord Rosebery), Lord Revelstoke, the Speaker of the House of Commons (Mr. J. W. Lowther, M.P.), the President of the Local Government Board (Mr. John Burns, M.P.), the Master of Elibank, M.P., Sir Ernest Cassel, Lieutenant-Colonel Sir Arthur Bigge, Sir Horace Marshall, Professor W. Osler, Mr. Vaughan Hawkins, Dr. Edwin Freshfield, Mr. J. G. Griffiths.

### Members of Finance Committee.

Lord Rothschild (chairman), Lord Revelstoke, Sir Ernest Cassel, Sir John Craggs, Mr. Robert Fleming, Mr. Hugh C. Smith.

### Members of Distribution Committee.

Sir William Church (chairman), Sir Trevor Lawrence, Sir Henry Burdett, Sir E. Cooper Perry, Sir John Tweedy, Mr. Frederick M. Fry, Canon Barnett, Sir Horace Marshall.

### Members of Convalescent Homes Committee.

Mr. William Latham, K.C. (chairman), Dr. Edwin Freshfield, Mr. Alfred Willett, Sir Walter Lawrence, Mr. S. H. Benson, Mr. George Lawson Johnston.

### Members of Executive Committee.

Lord Bessborough (chairman), Sir Savile Crossley, Mr. C. Stuart-Wortley, K.C., M.P., Sir William J. Collins, M.P., Mr. J. Danvers Power, Mr. A. E. Sydney, the Hon. Arthur Stanley, M.P., Mr. R. F. Cavendish, Mr. J. G. Griffiths, Mr. O. C. Philipps, M.P.

By the passing of the Act of Parliament we start, as it were, upon a new era in the life of the Fund. Without boasting, I think we may claim that our work has achieved great results. I hope it is fully realised by all who have given their valuable time and services, but more especially by the Honorary Secretaries and the members of the Executive and other committees, how heartily I recognise that it would have been absolutely impossible to secure these results without their zealous, unflagging work, and their self-sacrificing devotion to the interests of the Fund. With the same ready help, strengthened by that of our new members, I look confidently ahead. We have good friends who have given substantial proof of their belief in and appreciation of our work. The King watches over it with an interest no less keen than on the day of its foundation. As to myself, I never lose sight of the great responsibilities of our mission, and that it is our duty to direct our strenuous efforts towards the encouragement and support of efficient and economical administration of the London hospitals, and in this way to prove that we are worthy trustees of the funds committed to us by the charitable public, for the benefit of the sick poor of London. (Hear, hear.)

### How the Work will be Done.

On the motion of Sir William Collins, seconded by the Bishop of London, it was resolved that the following resolution be approved:—"That, until the General Council shall otherwise direct:—(*a*) The Finance Committee shall have the powers of the General Council with respect to the following matters—that is to say, the carrying into effect of sections 9, 10, 11, and 13 of King Edward's Hospital Fund for London Act—and such powers are hereby delegated to them. (*b*) The Distribution Committee shall have the powers of the General Council with respect to the following matters—that is to say, the rules and regulations to be observed with respect to applications from hospitals or other institutions for grants from the corporation, the determination as to which of such applications shall be considered, the making of inquiries into the circumstances and condition of any hospitals or other institutions applying for grants, and the making of recommendations to the President and General Council as to the distribution of the funds of the corporation to institutions not being convalescent homes, or such sanatoria for consumption as shall be situate outside the County of the City of London and the Metropolitan Police district, and as to all matters in respect thereof, and such powers are hereby delegated to them. (c) The Convalescent Homes Committee shall have the powers of the General Council with respect to the following matter—that is to say, the making of recommendations to the President and General Council as to the distribution of the funds of the corporation to convalescent homes, and such sanatoria for consumption as shall be situate outside the County of the City of London and the Metropolitan Police district, and as to all matters relating thereto, and such powers are hereby delegated to them."

On the motion of Sir Joseph Dimsdale, seconded by the Master of Elibank, it was resolved that the following resolution be approved :—" That, until the General Council shall otherwise direct, the Executive Committee shall have the powers of the General Council with respect to all matters not comprised in the foregoing resolution, save and except the expenditure of the funds of the corporation beyond a sum for the expenses of the administration of the Fund's general business, not exceeding £3,500, and save and except the making of rules and regulations under section 8 of the Act, and such powers are hereby delegated to them."

On the motion of Sir William Collins, the Council directed that it should be an instruction to the Executive Committee to consider and report upon the question of rules and regulations.

Other formal resolutions relating to banking account, etc., moved by Lord Rothschild, and seconded by Mr. H. C. Smith, were also approved.

The Lord Mayor moved, and Lord Revelstoke seconded, a vote of thanks to his Royal Highness the President, which his Royal Highness briefly acknowledged. The proceedings then terminated.

